# Prolonged lymphatic leak after retroperitoneal lymph node dissection: a case report

**DOI:** 10.4076/1752-1947-3-8704

**Published:** 2009-08-12

**Authors:** Katherine M Browne, Rowan G Casey, John A Thornhill

**Affiliations:** 1 Department of Urology, Adelaide and Meath Hospitals Incorporating the National Children's Hospital, Tallaght, Dublin 24, Ireland

## Abstract

**Introduction:**

Persistent lymphatic drainage following retroperitoneal lymph node dissection for testicular tumor is an uncommon complication.

**Case presentation:**

We describe a 21-year old man of Caucasian origin who had metastatic non-seminomatous germ cell tumor of the testis, and underwent retroperitoneal lymph node dissection, nephrectomy and partial inferior vena cava excision for a residual mass. The patient subsequently developed persistent lymphatic drainage causing foot drop that eventually responded to conservative medical and surgical measures.

**Conclusion:**

This postoperative condition usually responds well to conservative measures but has the potential for serious morbidity if it is not managed appropriately.

## Introduction

Persistent lymphatic fluid leakage is a rare complication of retroperitoneal lymph node dissection but has been described following a variety of vascular [1,2], gynaecological [3] and urological [4] procedures. It occurs due to disruption of the retroperitoneal lymphatics, which results in mechanical, nutritional and immunological dysfunction due to the constant loss of protein and lymphocytes. There are three major urological procedures identified in the literature which, when performed together or individually, are associated with refractory leakage of chylous fluid. These are nephrectomy, retroperitoneal lymph node dissection (RPLND) and inferior vena cava excision [5,6]. Our patient had to undergo all three procedures simultaneously.

## Case presentation

Following a right radical orchidectomy and four cycles of chemotherapy for a metastatic non-seminomatous germ cell tumor of the testis, our patient, a 21-year-old man of Caucasian origin, underwent RPLND for a residual mass. The procedure was prolonged and in order to achieve complete surgical excision, a right radical nephrectomy and excision of the inferior vena cava from the iliac veins to the level of the left renal vein was necessary due to tumor infiltration and encasement.

**Figure 1:**
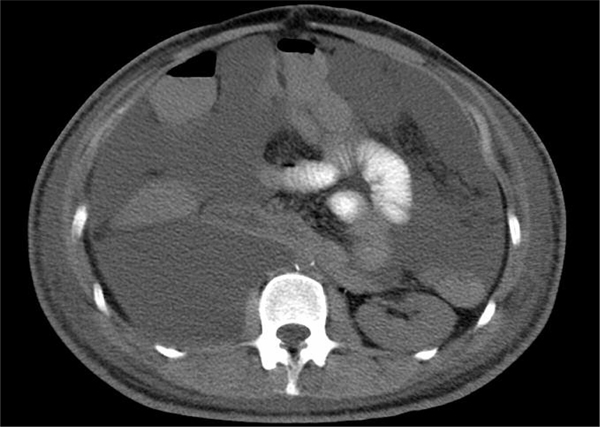
**A CT scan of the abdomen with an oral contrast agent, demonstrating marked intra-abdominal lymph leakage postoperatively with organ compression and abdominal compartment syndrome**.

**Figure 2:**
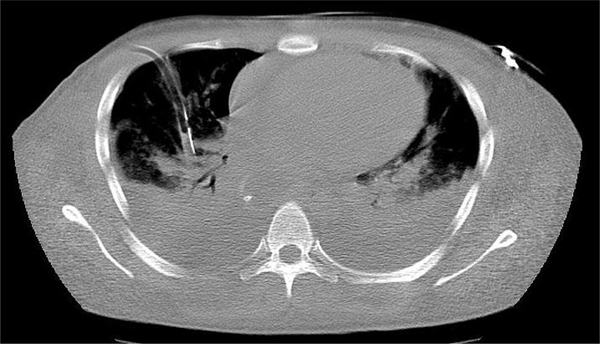
**A IV contrast enhanced CT scan of the thorax, demonstrating bilateral severe pleural effusions and right pleural chest drain insertion**.

Three days postoperatively, the patient developed bilateral leg pain, lower limb oedema and abdominal distension secondary to abdominal ascites (Figure 1). This continued to worsen and on day nine postoperatively, he developed a paralytic ileus, pleural effusions and respiratory failure, and was transferred to the intensive care unit for elective intubation, ventilatory support and chest tube drainage of the pleural effusions (Figure 2).

With fears that our patient would develop intra-abdominal compartment syndrome and that the respiratory compromise would be worsened, a 12 Fr pigtail drainage catheter was inserted under ultrasound guidance on postoperative day 22 into the abdominal cavity, immediately producing 11 litres of chylous fluid. Shortly after a drain was inserted, the respiratory compromise improved and the patient was extubated. The drain continued to maintain an output of six liters per day for a further four days before settling to an average output of between two and three liters per 24 hours. Conservative management consisted of a parenteral diet of medium chain triglycerides, diuretic therapy and 20% albumin three times a day. Daily intraperitoneal infusion of 200 mls of the water-soluble contrast medium Conray 280 mg I/ml (Iothalamate meglumine) in order to promote peritoneal lymphatic fibrosis was attempted but produced no real improvement. Blockage of the intra-abdominal drain necessitated its replacement on two separate occasions.

On day 41, the patient was discharged to the ward where he maintained this improvement. Total parenteral nutrition was commenced to supplement poor oral intake. The patient had also developed bilateral foot drop on day 22 postoperatively. This was felt to be due to pedal oedema compressing each peroneal nerve at the head of fibula. It improved gradually with resolving peripheral oedema and physiotherapy. He was discharged to a peripheral hospital for further care on day 53, with an average output of 1.5 liters. Histologically, all the resected retroperitoneal tissue was free from residual tumor and contained only lymphoid tissue with large areas of necrosis.

## Discussion

This post-surgical complication can be life-threatening and has a relatively high morbidity and mortality rate if not managed correctly. It may potentially result in fluid and/or electrolyte imbalances, severe malnutrition, lymphopenia and overwhelming infection. However, in the absence of any underlying malignant or congenital pathology, the prognosis in cases of postoperative persistent lymphatic leakage is good, with the majority responding to conservative measures. These consist of total parenteral nutrition, a diet of medium chain triglycerides, diuretics and more recently, the use of somatostatin analogues especially in refractory cases [7,8]. Surgical management is limited and consists of repeated paracentesis, abdominal drain insertion and surgical closure of the lymphoperitoneal fistula. Several older case reports supported the use of surgical peritoneovenous shunting procedures (Denver, LeVeen), especially in prolonged high output leakage [5,9]. When a peritoneovenous shunt is required, it may be needed for an extensive period for resolution and there are significant complications associated with its use [10].

## Conclusion

In this case, the patient was managed in a conservative fashion. However, insertion of an abdominal drain was eventually required to relieve abdominal compartment syndrome. The use of Conray in this case was not effective or justified as it is water-soluble and does not promote significant peritoneal fibrosis [11]. The potential for serious deterioration with this postoperative complication was demonstrated with requirement for ventilation and subsequent foot drop in this patient. Overall, prognosis should remain good once these rare postoperative sequelae are recognized and if treatment is commenced in a timely fashion.

## Abbreviation

RPLND: retroperitoneal lymph node dissection.

## Consent

Written informed consent was obtained from the patient for publication of this case report and accompanying images. A copy of the written consent is available for review by the Editor-in-Chief of this journal.

## Competing interests

The authors declare that they have no competing interests.

## Authors' contributions

KB analyzed and interpreted the patient data, reviewed the notes and wrote the first draft of the report. RG rewrote the manuscript and assisted in the literature review. JT performed the presentation, was the chief clinician and was the major contributor in writing the manuscript. All authors read and approved the final manuscript.
